# Home care for patients with dirty homes: a qualitative study of the problems experienced by nurses and possible solutions

**DOI:** 10.1186/s12913-022-07988-2

**Published:** 2022-05-03

**Authors:** Anke J. E. De Veer, Kim De Groot, Renate Verkaik

**Affiliations:** 1 Netherlands Institute for Health Services Research (Nivel), PO Box 1568, 3500BN Utrecht, The Netherlands; 2 Thebe Wijkverpleging (Home Care Organization), Lage Witsiebaan 2a, 5042 DA Tilburg, The Netherlands

**Keywords:** Self-neglect, Home-care nurses, Hoarding, Person-centred care, Integrated care

## Abstract

**Background:**

Home-care nurses are often the first care professionals to enter a dirty home. The perceived problems and support needs of home-care nurses in these situations are largely unknown.

**Objective:**

To examine the problems home-care nurses encounter in caring for patients living in dirty homes, and possible solutions for these problems.

**Design:**

Qualitative descriptive research.

**Setting:**

Communities across the Netherlands.

**Participants:**

Twenty-three participants to investigate the problems or needs experienced, and 20 participants to investigate solutions. Participants included patients, home-care nurses and other professionals working in the community.

**Methods:**

Semi-structured interviews were conducted with 23 participants and analysed according to the principles of deductive thematic analysis. Subsequently, in interviews with 4 (representatives of) patients and four focus-group sessions with 16 professionals, the problems found were validated and solutions to the problems discussed.

**Results:**

Ten subthemes emerged that were clustered into three main themes: ‘dilemmas arise in choosing the right nursing care’; ‘cooperation and an integrated approach are often necessary, but lacking’; ‘home-care nurses have insufficient competencies’. Seven possible solutions were found: (1) strengthening collaboration between organizations in the community; (2) involving others sooner; (3) case management; (4) person-centred care; (5) taking more time; (6) providing home-care nurses with tools and support services; and (7) strengthening the competencies of nurses.

**Conclusions:**

Care for patients with a dirty home is complex. An integrated person-centred care approach is often necessary and home-care nurses need extra support to provide such care. Interventions should not only focus on patients, but address the nurses, the organization, and the collaboration between organizations in the community.

## Background

Although there is no clear definition of a dirty home, a dirty home entails health and safety risks for the residents and their neighbourhood. Health risks include the risks of infection, food poisoning and respiratory problems due to an excess of mould or bacteria. Examples of safety risks are the risk of falling and fire. Most health-care professionals do not visit their patients in their own home environment, but receive them in the professional’s practice. Home-care nurses are often the first health-care professional to enter the home, for example after a patient’s discharge from hospital or after a general practitioner’s referral. Home-care nurses therefore play a critical role in identifying possible risks and underlying causes and organizing further support and care for patients with dirty homes. This article describes the problems nurses experience in the care for patients with a dirty home and the possible solutions to these problems.

In the Netherlands, as in many other European countries, home-care nurses provide nursing care and personal care in people’s homes [1]. Nursing care can be of a technical, supportive, rehabilitative or preventive nature. Personal care refers to assistance with activities that are part of everyday life, such as dressing, feeding and washing.

Dirty home conditions can make it difficult or even impossible to provide nursing or personal care. If patients do not want to, or are not able to, tidy up or clean their houses, home-care nurses should decide what the right thing is to do for the health and wellbeing of these patients, as well as for their family, the neighbourhood and the health of the nurses themselves.

Although entering an extremely dirty home is considered a problem by many nurses, scientific research into the tasks of nurses in these situations is scarce [2]. Empirical studies among nurses describe a dirty home mainly as a symptom of self-neglect in elderly people [3–6]. Self-neglect behaviours, such as neglect of the home, lack of personal hygiene or refusing help, were found to result in feelings among nurses of confusion and ambiguity regarding the nurses’ role [2].

However, a dirty home does not just happen in old age. In general, three situations can be distinguished. Sometimes someone is temporarily unable to take care of their own home, for example because of illness or grief. Secondly, a dirty home can be a sign of decreasing functional or cognitive functioning [3, 4], such as dementia. And thirdly, there is a group with a variety of (often interacting) causes and problems [3, 7]. Associations are found with intellectual disabilities, lower socioeconomic status and alcohol dependence [3, 8]. In addition, social isolation and loneliness play a role [9]. A dirty home can also be a sign of psychiatric problems, such as schizophrenia and depression. Confusion, inactivity or indifference can cause a person to neglect the home. Hoarding can also lead to a dirty home. In the Diagnostic and Statistical Manual of Mental Disorders of the American Psychiatric Association (DSM-5), a hoarding disorder is a psychiatric diagnosis characterized by persistent difficulty discarding or parting with possessions, regardless of their actual value [10]. Some people may have hoarding issues which make the delivery of care difficult, but the home itself is not actually dirty. Hoarding leads to a dirty home if the home is so cluttered that cleaning is no longer possible. Given the variety of possible underlying causes and problems, the challenge for home-care nurses is to respond adequately, and appropriately, when faced with patients with dirty homes.

With the increase of life expectancy and the general deinstitutionalization policy in the Netherlands and many other European countries, an increasing number of the elderly, people with psychiatric problems and people with intellectual disabilities live independently in the community [11]. Since home-care nurses often perceive entering a dirty home as a problem, we aim to explore the issues they face in providing care for these patients and how these issues can best be solved. The research questions are:

1. What problems do home-care nurses face in the care for people living in dirty homes?
2. What are possible solutions regarding the problems in the care for people living in dirty homes?

## Methods

### Design

A qualitative research design was used including individual interviews and focus-group discussions [12]. First, qualitative interviews about the experienced problems or needs of home-care nurses were held with people with dirty homes or their representatives, and professionals who provide support and care to people with dirty homes. Second, solutions to the problems were discussed in focus-group sessions. Due to their interactive nature, focus groups can produce valuable solutions that are less easily found through individual interviews. Initially, we planned five focus-group sessions: one with people with a dirty home or their representatives, two with a mixture of professionals in health care and social care, and two with home-care nurses. All four focus groups with professionals were video-conferencing sessions. Because of the COVID19 pandemic, the focus group with patients and their representatives could not take place on location. This focus group was replaced by individual interviews by phone. Each focus group started with a reflection on the problems mentioned in the interviews in order to assess the validity of the described problems. Subsequently, possible solutions to the problems were discussed. The interviews with patients and their representatives focused on the kind of support they wished to receive.

### Recruitment and participants

The participants were recruited by convenience sampling. Professionals had to meet the inclusion criterion of having experience with people in dirty homes. As mentioned in the introduction, a variety of problems and causes may underlie a dirty home. Therefore we interviewed not only home-care nurses, but also other professionals with experience of people with dirty homes, for social workers, in order to get a rich picture of the possible problems and perceived solutions. To increase the variety of experiences, we strived for variation in participants’ characteristics in terms of: type of organization (e.g. health care, social care), region in the Netherlands (north, east, south, west), and urbanization (e.g. village, town). People with a dirty home and their representatives were recruited by an interest group for people with psychiatric problems, by an interest group for the elderly and by the professional participants.

Participants received verbal information and an information letter and signed an informed consent form. Participants were provided with a €50 gift voucher.

### Data collection

The interviews were face to face, by phone or video call, depending on the preference of the interviewee. Interviews were conducted in July, August and September 2020 by one of the two researchers (AdV, RV). AdV and RV were senior researchers and both trained as psychologist. KdG had an advisory role in the collection and analysis of data and was a researcher and home-care nurse, trained as RN and Master of Nursing Sciences. The interviews lasted between 45 and 60 min.

Open questions were used, encouraging interviewees to describe their problems and needs in their own words. Table 1 shows the interview guide with key questions used in the interviews with patients and home-care nurses. Similar interview guides were used for the interviews with representatives of patients and the other professionals, respectively. Professionals not working in home care were additionally asked to reflect on their experiences with home-care nurses and the problems home-care nurses faced.

**Table 1:** Key questions and examples of additional questions for the interviews with patients and home-care nurses

Interview with patient
1. *Can you tell us something about yourself?* (age, living situation, region, health)
- What does your home look like?
- Has the home always been as it is now or is it something of the last few months?
- What care do you receive from the home-care nurse? How often?
- Can you tell us more about your experiences with this care?
2. *Do you ever find it difficult when home-care nurses come into your home?* When?
3. *If you find it difficult, what has this got to do with?*
- How do you feel when a home-care nurse makes comments about what your home looks like?
- Can you imagine that the home-care nurse finds it difficult to provide care in your home?
- Has a home-care nurse ever refused to provide care due to the situation in the home?
4. *How can the home-care nurse make things more comfortable for you? What is needed for that?*
*5. What help do you need when it comes to keeping the house clean or collecting things safely?*
- How would you feel if the home-care nurse were to seek help from others in connection with the situation in your home?
- Do you dare let other people into your home?
**Interview with home-care nurse**
1. *Can you tell us something about yourself?* (profession, organization, region)
- Can you tell us more about your experiences with patients with a dirty home?
- When do you call a home dirty?
- Have you ever refused to provide care?
2. *What problems do you run into when providing care in a dirty home?*
(Think of care-related or organizational matters, own competencies, concerns about own health, etc.)
3. *Which factors play a role in the problems you mentioned?* (Think of the patient, the system around the patient, home-care nurses, home-care organization, other professionals or organizations.)
4. *What is needed to facilitate home care for these patients?*
5. *What care do you think patients need when it comes to their dirty homes?*

The focus-group sessions were held over a period of three weeks to allow the researchers to take possible solutions that had been put forward in one session and propose them for discussion in subsequent sessions. Because we aimed to find solutions for home-care nurses, we started with two focus groups with the other professionals (*n* = 9) and the interviews with patients and their representatives (*n* = 4) (Table 2). The final two focus groups were with home-care nurses (*n* = 7). Each focus-group session was moderated by a researcher (AdV) while another researcher took notes (RV). Each group session lasted two hours. First the results of the analyses of the problems identified in the individual interviews were presented and discussed. The participants found the problems were very recognizable, indicating that the list of problems was valid. Subsequently three questions were discussed: (1) What are solutions for these problems? (2) Which solutions are most promising and should have priority? (3) What are examples of good practices? In the final 30 min of the session the researcher (RV) summarized the solutions and participants were asked to reflect on this summary.

**Table 2:** Characteristics of the phase 1 interviewees (*n* = 23) and phase 2 participants in the focus groups (*n* = 16) and individual interviews (*n* = 4)

Phase 1 participants (research question 1)	Total *n* = 23	Phase 2 participants (research question 2)	Total *n* = 20
**Type of participant**		**Type of participant**	
- patients	*n* = 2	- patients	*n* = 1
- patients’ representatives (family, other informal carers)	*n* = 3	- patients’ representatives (family, other informal carers)	*n* = 3
- home-care nurses	*n* = 8	- home-care nurses	*n* = 7
- public-health nurses in the community (social support and prevention)	*n* = 5	- public-health nurses in the community (social support and prevention)	*n* = 4
- nurses in mental health-care organization	*n* = 2	- nurses in mental health-care organization	*n* = 4
- social workers in social housing association	*n* = 1	- social workers in social housing association	n = 1
- general practitioners	*n* = 1		
- social workers in the community	*n* = 1		
**Region in the Netherlands**		**Region in the Netherlands**	
- north	*n* = 3	- north	*n* = 2
- east	*n* = 5	- east	*n* = 4
- south	*n* = 4	- south	*n* = 3
- west	*n* = 11	- west	*n* = 11
**Urban environment**		**Urban environment**	
- large cities (Amsterdam, Rotterdam, The Hague and Utrecht)	*n* = 8	- large cities (Amsterdam, Rotterdam, The Hague and Utrecht)	*n* = 7
- other urban	*n* = 4	- other urban	*n* = 4
- rural	*n* = 11	- rural	*n* = 9

### Analyses

The individual interviews were audiotaped and transcribed verbatim. Transcripts were returned to participants for correction. The analysis was part of a cyclical collaborative process of data collection – data analysis – new data collection by two researchers (AdV and RV), ultimately ending in data saturation [13]. Transcripts were analysed qualitatively according to the principles of thematic analysis [14]. All transcripts were analysed by AdV and RV, where AdV analysed the interviews held by RV and vice versa. First the researcher read a transcript to become familiar with the content. In the next steps initial codes were given while staying semantically close to the text. The next steps included searching for underlying themes and checking whether the themes worked in relation to the codes and dataset. This procedure of naming underlying themes and checking was repeated several times until the researchers reached consensus. Finally, the themes were described. The software program MAXQDA (release 11.0.9b) was used to facilitate the process of data analysis [15].

The individual interviews about solutions were transcribed verbatim. Transcriptions were also made of the final 30 min of the focus-group sessions. After the interviews and each focus-group session, researchers searched for the underlying possible solutions, to finally reach consensus.

## Results

### Participants

Individual in-depth interviews about the problems of home-care nurses in dirty homes were held with patients and their representatives (*n* = 5), home-care nurses (*n* = 8) and other professionals in the community who deal with people with dirty homes (*n* = 10), with a good spread across different regions and communities (Table 2). Twenty participants with comparable backgrounds to the initial interviewees discussed how best to deal with the problems (Table 2).

Problems home-care nurses face

**Fig. 1:**
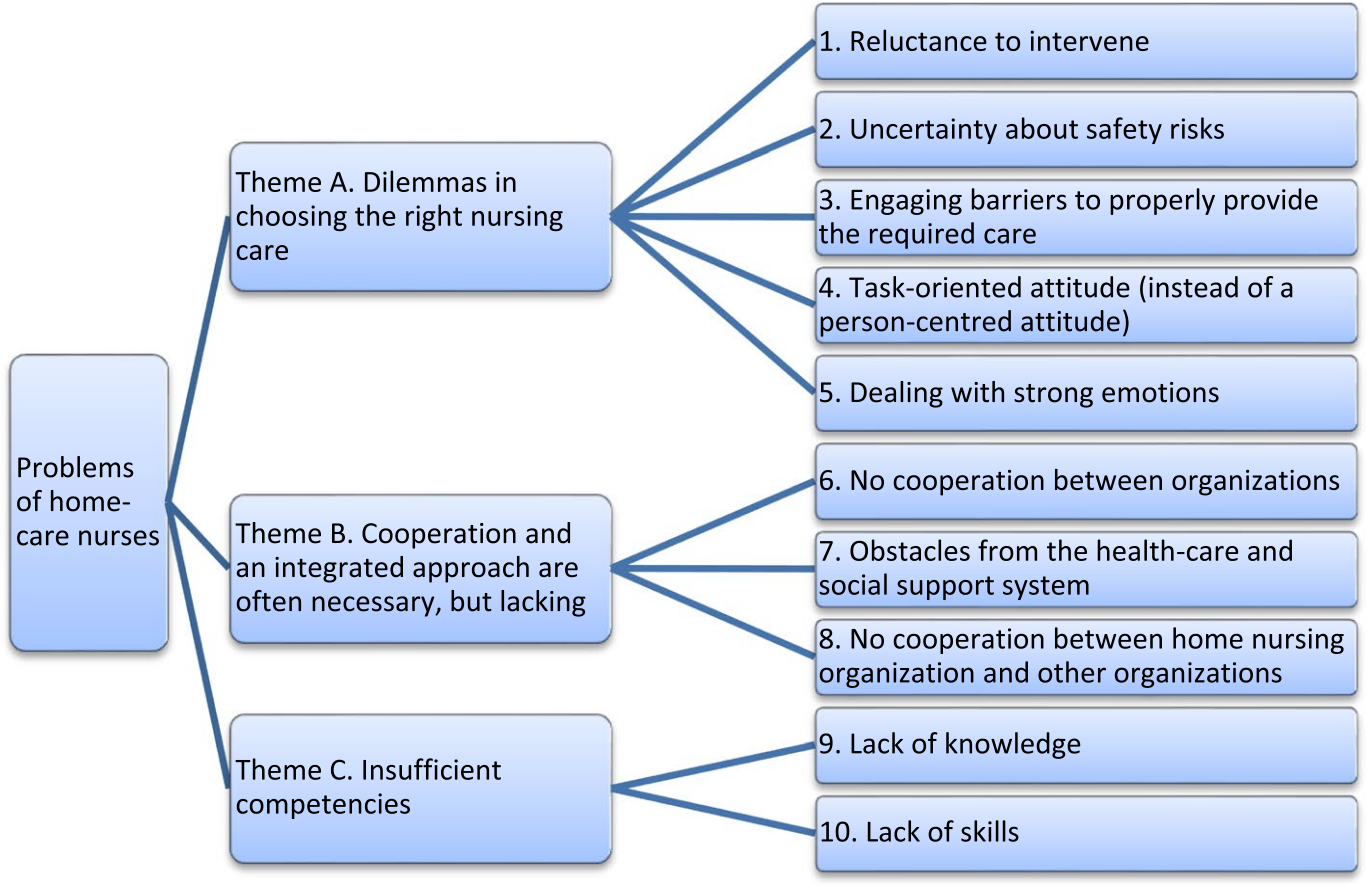
Three main themes and ten subthemes characterizing the problems faced

The interviews revealed three main themes and ten subthemes in the problems that were mentioned regarding the provision of home care for people with a dirty home: (A) there are dilemmas in choosing the right nursing care; (B) cooperation and an integrated approach are often necessary but lacking; and (C) nurses have insufficient competencies (Fig. 1).

A. Dilemmas in choosing the right nursing care

Five subthemes form this main theme about what kind of care home-care nurses should provide to patients with a dirty home. Home care nurses were *reluctant to intervene. They did not want to involve colleagues and professionals* from other organizations because they first wanted to win the trust of a new patient. Home-care nurses who had built a relationship of trust after a period of time were anxious about losing the patient's trust because they were afraid that the patient did not want them to turn to someone else for help. As a public-health nurse specialized in dealing with the inhabitants of unclean houses remarked: *“Home-care nurses sometimes take care of someone for years, so there is a lot of trust there. They sometimes do more than their duties. And then they're afraid of losing the patient's trust because the patient doesn't want her to get someone else's help. They have, as it were, penetrated the patient's system and can no longer maintain a professional overview*”.

Home-care nurses were also *uncertain about the safety risks and how to deal with them* in a dirty home. They found it difficult to estimate the risks and dangers: When is the house safe enough for the patient, the environment and myself? There were generally no frameworks, decision instruments, or guidelines on when and how care could be refused and what the next steps were. Another issue that emerged was that norms and values about what constitutes a dirty home differed between home-care nurses: “*Some find a lot of dust or cigarette smoke problematic; others don’t care*”. Nurses found it difficult to determine how to provide care in a dirty home. For example, some sat on a plastic bag for protection or left their coat and bag in the car. Others showered at home before moving to the next patient or scheduled the patient at the end of the shift.

The third subtheme concerns *barriers to properly providing the required care*. On the one hand, this had to do with the inability to provide care safely and hygienically, such as providing wound care in the presence of a lot of dust, dirt or vermin. As a nurse specialized in wound care said, ”*A lot of specialist care is provided. And also care where you really have to be able to work hygienically. Usually it is possible to create a clean work field somewhere, but in such a very dirty household I find that difficult*”. Another problem was helping someone with showering if the shower was not accessible or if there were no clean towels. Care provision was also perceived as less feasible because of the extra time required. It took more time to provide care because of the extra preparations required, such as clearing things to make room and cleaning a surface. It also took time to gain the patient's trust and discover possible underlying problems. Therefore additional time was needed, which was not always available in tight timetables.

A fourth subtheme that arose was a *task-oriented attitude* among nurses. Nurses with a task-oriented attitude strived to provide the indicated care and support. A person-oriented attitude is required in particular for patients with dirty homes. The task-oriented attitude was sometimes evident in, among other things, posting indirect and judgmental comments about the house and being patronizing to patients. As a psychiatric nurse in the community remarked, “*What we sometimes notice is that things don't go well within regular home nursing, for example because they then come in and they immediately have to have a high-low bed. And the rug has to go, because otherwise the bed will roll too heavily. Yes, they immediately get a whole bunch of garbage bags that have to go and that trash can is not good, neither is that shower drain. If people do have a certain disorder, it is often far too much and they start screaming or cursing. ‘Get out of my house! What are you meddling with?’ And then they slam the door*”. Home-care nurses often focused on what they could *not* do in a dirty house, rather than looking at what it was still possible for them to do.

Finally, care provision in a dirty home was hindered by the *strong emotions* that the situation evoked in nurses, such as fear and feeling dirty, sometimes even leading to panic. There were also feelings of helplessness: nurses worried about patients and their situations, but did not know what to do about it. Nurses were also concerned about their own health. Sometimes the contamination was so intrusive that nurses mainly saw the dirt and not the patient: the dirt and clutter were overwhelming, as a home care nurse explained: *“Really accumulated dirt, accumulated dust, grease. And I remember going to the kitchen to get a dustpan, and then I came across a fly trap, such a sticky thing. Which was also completely covered in flies. I got them in my hair. And then I panicked. And then I know the client said, ‘Oh, that's okay girl’. But luckily I still had my gloves on, so I forcibly took them off because I thought it was so disgusting. I found it really disgusting. But I see things like that regularly”.*

B. Cooperation and an integrated approach are often necessary but lacking

Three subthemes concerned barriers to cooperation and an integrated care approach (Fig. 1). Participants in the focus groups confirmed that the most prominent problem was the *lack of cooperation between different organizations* in the community, such as home-care organizations, public-health services, welfare and housing. A dirty home was often the result of complex problems such as recurring psychological, psychiatric, physical and/or cognitive problems, a limited social network, financial problems and general avoidance of care. A new home-care patient was often already known to professionals in other organizations. But, as a home-care nurse remarked, “*Collaboration is crucial, but there is a lack of coherence in the care provided. They are all islands*.” According to the interviewees, this was where things went wrong because organizations did not have cooperation agreements, and professionals did not know each other and were not aware of each other's possible roles in the care for patients with dirty homes. The public-health service sometimes had a team specialized in dirty home conditions, including social nurses with a lot of knowledge. However, other professionals in the community were often unaware of this.

Integrated care was also frequently hindered by the design of the Dutch *health-care and social support system* (structure, laws and regulations)*.* Participants mentioned the de-institutionalization of care in recent years, in particular for the elderly, people with psychiatric problems and people with intellectual disabilities: people now live independently in the community as much as possible and for as long as possible. Care and support facilities were rather fragmented and integrated care was found to be difficult to achieve. For example, there was a division between domestic care and social support financed by the municipality on the one hand and the nursing care delivered by home-care nurses under the Health Insurance Act on the other hand. There were also local differences in facilities, for example in whether the municipality contributed to the financing of the cleaning of dirty homes.

A third subtheme focused on the role of home-care organizations and the *lack of cooperation between home-care organizations and other organizations*. Home-care nurses rarely contacted other organizations in the fields of health care, welfare and housing regarding patients with dirty homes. A psychiatric nurse pointed out that whenever a nurse contacted a psychiatric organization, “*that always takes a lot of time. Because then you have to do the intake, and there are waiting lists and such. And if home-care organizations have to provide physical care in the meantime, the nurse is alone. And then the nurse has to deal with very complex psychiatric problems*”. Problems with the coordination between home-care organizations and hospitals or general practitioners were also mentioned: hospitals sometimes did not accept patients because of physical neglect and sent them back home without consulting home-care nurses. Or a hospital discharged a patient without consulting the home-care organization, even though they knew that adequate home care was not possible. According to the interviewees, general practitioners also regularly said that if a patient chose to live in a dirty house, this was his or her own choice.

C. Insufficient competencies

Insufficient competencies of home-care nurses were also mentioned as problematic (Fig. 1). When it comes to *knowledge*, interviewees agreed that home-care nurses lacked knowledge about the potential underlying problems of dirty-home patients. This had to do with the often complex, multiple problems, which sometimes went on for years and could originate in a patient’s youth. For example, it could be cognitive or functional decline, psychiatric problems, but also addiction, grief, debts or combinations of different problems. As a social worker explained, that knowledge is needed to recognize the seriousness of the situation: “*I think there is a bottleneck there in the knowledge about people with psychiatric problems… often chronic or addiction problems of the same kind. Yes, that's a problem that won't go away or can’t be solved so easily”.* Lack of knowledge made it difficult to know when to engage professionals from other organizations. As mentioned earlier, lack of knowledge also applied to knowledge about the services available in the community. Even if there was a team within the community that provided assistance to people with dirty households, home-care nurses usually were not aware of this. Another knowledge gap concerned the possibilities of discussing a patient with a dirty home with professionals in other organizations within the boundaries of privacy legislation. According to the interviewees, a lack of knowledge prevented home-care nurses from discussing their feelings and doubts with colleagues and contributed to stigmatizing patients within the home-care team.

Home-care nurses also generally lacked *skills* to adequately respond to patients with a dirty home. They found it difficult to motivate patients to clean their houses. They also found it difficult to respond if it turned out that patients had not cleaned up their home sufficiently despite agreements that had been made. Another skill that was often lacking was an ability to discuss the patient’s situation with colleagues without prejudice.

### Solutions

The focus-group sessions with professionals and the individual interviews with patients and their representatives revealed seven possible solutions (Table 3). First, there was a general plea to *strengthen the collaboration between organizations* in the region. This was seen as a prerequisite for providing personalized, integrated care, which was considered necessary to provide good quality care to patients with dirty homes. Due to the differences between regions (Table 3), cooperation agreements and the coordination of care are best made at a community level. All relevant parties should be represented, including at a minimum the public-health services, the municipality, organizations for psychiatric care, organizations for domestic care, general practitioners, hospitals, housing corporations, police and the fire brigade.

**Table 3:** Solutions letting home-care nurses and their organizations provide adequate support and care to patients with a dirty home

1. Strengthening collaboration between organizations in the community
2. Involving others sooner
3. Appointing a case manager
4. Shifting from task-related care to person-centred care
5. Allocating and taking more time
6. Giving home-care nurses tools and support services
7. Increasing the competencies of nurses

Second, home-care nurses should *consult other professionals sooner*. Patients, representatives of patients, and professionals emphasized the important role of home-care nurses in identifying problems with a dirty home and in quickly calling in other professionals. They emphasized that home-care nurses often visited a patient when the condition of the home had clearly been bad for a long time. It often made little sense to wait and see if the patient started to tidy up. The recommendation from professionals in the focus-group discussions was "*Dare to act*". Home-care nurses should share their concerns with colleagues. The general advice was to quickly contact a central reporting point in the community. There should always be at least a central telephone number and ideally there should be a specialized team with knowledge and expertise about dirty homes, where a home-care nurse can anonymously discuss a patient or ask someone from the team for a joint home visit. An experienced professional can often assess properly what is going on and what follow-up steps can be taken. An advantage of this can also be that in extreme cases the experienced professional can suggest the need for cleaning or additional help, while the patient's relationship of trust with the home-care nurse remains.

In addition to the first two recommendations, it was emphasized that a *case manager* is needed to arrange multidisciplinary and inter-organizational collaboration at the patient level. All professionals who are already involved with a patient should be consulted and should discuss, among other things, the safety of the situation, the underlying problems and the possible solutions and appropriate help, in part to prevent a recurrence of the situation. For this it is necessary that someone coordinates the care. According to the focus-group participants, the professional who is best placed to fulfil this role depends on the specific situation of a patient. Sometimes the home-care nurse can take the lead (for example in the case of elderly people with physical problems), in other cases a psychiatric nurse is more appropriate (for example in the case of hoarding) or a nurse or social worker from the public-health service (for example if a patient refuses help which proves necessary due to safety risks).

A fourth recommendation is to provide *person-centred care*, that is to say: focus on the wishes and needs of the patient. It was emphasized that it is important that the patient sees the same professionals with whom the patient gets along well. New professionals should be introduced where possible by a trusted professional, preferably the case manager. First of all, work must be done on building a relationship of trust with the patient. To gain confidence, it is important to take enough time to talk to the patient, to listen and to be empathetic. The dirty home conditions and the possible risks must be mentioned, but without judgement. At the same time, home-care nurses can indicate that they want to help think about possible solutions. It is a search for the right balance: some urging can sometimes also be a relief for a patient because his or her situation will change. Since the condition is usually not resolved overnight, home-care nurses also have to think ‘outside the box’, for example, by looking at whether it is possible to shower in a community centre or to provide wound care in a general practice.

The professionals agreed that *extra time* should be allocated to be able to provide person-centred care. Extra time is needed to get to know the patient and gain his or her trust. The care itself often also takes more time, for example if a patient does not cooperate or when time is needed to find or create a place to provide the care. Investigating the underlying problems, consultation within the home-care organization and collaboration with professionals from other organizations also require extra time.

Furthermore, the development and use of *tools and support services* help home-care nurses provide integrated person-centred care to patients with dirty homes. The professionals emphasized that these tools and support services only help if they are supported by the management of the home-care organization. Employers should take a clear position when it is not possible to deliver care in a particular situation, and support the nurses in this. Every home-care organization should have a step-by-step plan detailing what home-care nurses can do when the house is too dirty. Such a roadmap should contain tools to describe the level of dirtiness (such as the Clutter-Hoarding Scale, [16]), criteria for giving and refusing home care (for example answering the question, “Can I do my job as intended?”) and a map of organizations in the community. Another type of tool supports collaboration with all the people involved. Examples are a cross-organizational communication app, a list of all professionals and family members involved with the patient, or a sociogram to map and involve the patient's social network. In addition, there should be a person in each home-care organization who can be consulted by home-care nurses if they have a patient with a dirty home. This person knows the procedures and the agreements with other organizations and can refer home-care nurses to an external expert. This person can possibly pay a home visit to assess the situation. If necessary, this person can also organize lessons by, for example, the public-health service, psychiatric services or the fire brigade.

Finally, the *competencies* of home-care nurses should be increased. The professionals agreed that the problem of dirty households is too complex and too rare to train nurses to become experts. Nurses should nevertheless have basic knowledge and skills about people with dirty households. This should cover, among other aspects, the possible underlying problems, the right approach and relevant legislation and regulations in the field of privacy.

## Discussion and conclusion

Home-care nurses faced three clusters of problems in the delivery of home care to patients in a dirty home: dealing with dilemmas in choosing the right nursing care, lack of inter-organizational collaboration, and insufficient competencies of home-care nurses. Giving home care to patients in a dirty home while having *doubts about the best strategy* and feeling unskilled is demanding for home-care nurses. Dirty home conditions were found to evoke strong negative emotions in nurses, which is also described in previous literature [2, 6]. The overwhelming emotions blocked the ability to empathize and respond with compassion to the patient. Nurses could not see the needs of the patient anymore. A meta-analysis has shown that nurses’ stress is negatively associated with compassion [17], and this influences the ability to focus on the patient’s needs and the quality of care. Nurses were found to experience dilemmas. These dilemmas related to whether or not to intervene, how to deal with possible safety risks, how to perform the required interventions, and choosing between a task-oriented and person-oriented attitude. Nurses reported similar dilemmas in cases of self-neglect among older adults [5]. In addition, patients stressed that professionals should respect their way of living. A qualitative study by Day et al. [18] among a small group of elderly patients found that most participants were content with their way of living, and thought decisions to live this way must be respected.

A second cluster of problems consisted of *barriers in collaboration between organizations*. Both professionals and patients and their representatives made a plea for *integrated* person-centred care. This desire on the part of patients was also found in other studies [19]. An integrated person-centred approach was hindered by a lack of collaboration between organizations and the silos in the health-care and support system. Whilst the focus of the current paper is on issues relevant to the Netherlands, the perceived barriers due to the health-care system, such as different funding and separate services, is not unique and is also experienced in other European countries, Australia and Canada for instance [20–24]. For example, in many European countries domestic care is part of the community-based support system [1, 11].

The third cluster of problems was a *lack of basic competencies* among home-care nurses. A wide variety of patients can have dirty homes, such as the elderly, people with psychiatric problems and people with intellectual disabilities with a lot of different underlying problems. Therefore there is no standardized solution to help them. Although home-care nurses are used to improvising, which they generally perceive as an attractive aspect of their job [25], this was often not the case with patients in dirty home conditions. Insufficient knowledge and communication skills exacerbated the dilemmas faced by home-care nurses. Education about possible causes, local policy and care pathways, privacy issues (such as involving other professionals without permission), and legislation regarding how to deal with support refusal (such as trespassing without permission) are also mentioned by others as important areas of knowledge for nurses [3, 26]. Findings on knowledge about self-neglect among the elderly showed that the majority of nurses working in the community had not had any education on the subject of self-neglect [27]. As we also found in our research, building a relationship of trust, being non-judgmental and respecting the autonomy of patients are important basic skills [5, 28].

These problems show that in terms of the Job Demands-Resources model [29], people with dirty homes are found to put many demands on home-care nurses. There is no balance between the demands placed on home-care nurses and the available resources. The Job Demands-Resources model helps the professional to reduce job demands by enhancing resources to adequately deal with the demands the dirty home conditions pose. More resources can be found at the level of the health-care system, the home-care organization, and the individual home-care nurses.

It was concluded that people with dirty homes need an integrated person-centred care approach. This cannot be attained by focusing exclusively on the individual nurses and the home-care organizations but requires reforms eliminating the silos within the *health and social care system*. The World Health Organization [30] pointed out that in many countries fundamental shifts in the planning and delivery of health services are needed. Such policy reform proposals require a whole-of-government approach across silos. Governments are reluctant to undertake such reforms due to the multitude of changes required in policy jurisdiction, financing and governance complexities [31]. The professionals in our study considered community-based collaboration a more feasible strategy to attain inter-organizational collaboration. Although the effectiveness of integrated care for patients has not been proven [32], a systematic review showed that there is evidence that integrated care leads to better care processes such as documentation and referrals [33]. Our research indicates that integrated person-centred care may benefit the working conditions of home-care nurses because of the perceived support of other professionals and better care processes.

*Resources within the home-care organization* that were mentioned as helpful were providing home-care nurses with tools and support services and enabling case management. The home-care management should provide tools such as a step-by-step roadmap that outlines what nurses can do. Such a roadmap could contain, among other things, advice on how to respond, possibilities for action while complying with privacy legislation, a list of roles and addresses of experts, criteria to assess the situation, and a tool to describe the patient’s network. Furthermore, there should be an expert or an expert team for consultation within each organization.

Case management is generally considered as a core component of integrated care to attain co-ordinated care and continuity of care [20, 34, 35]. The home-care organization should also give nurses enough space to collaborate with colleagues and professionals outside their own organization. Koenig et al. [36] evaluated multidisciplinary teams in Kansas (USA) helping older adults with hoarding problems. For each case they formed a task force with members from different organizations to meet the needs of the patient. Four components for successful team work were found: (1) team members working together to address issues such as role conflict or a lack of expertise, such as no mental health providers in a team; (2) consistency in organizational policies that define the scope or extent of a team member’s involvement; (3) sufficient external support such as funding for extra mental health services or local publicity; and (4) the team members’ capacity to develop trust with patients.

Finally, we found a requirement for resources at the *level of individual home-care nurses*: basic knowledge and skills, extra time to build a relationship of trust with the patient, and engaging other professionals at an early stage. Research showed that most home-care nurses regard the different aspects of integrated person-centred care as attractive, but difficult to attain [37, 38]. Nurses were generally reluctant to intervene and consult other professionals. They wanted to respect the patient’s autonomy and freedom to choose how to live. They also were unwilling to intervene because they did not want to risk their relationship of trust with the patient. In addition, nurses were afraid that calling in others would violate the General Data Protection Regulation (GDPR). More basic knowledge and skills can help the nurse to respond adequately and arrange integrated person-centred care sooner.

### Strengths and limitations

To our knowledge, this is the first study of the provision of home care for people with a dirty home. It addresses a situation that is felt to be extremely difficult by many home-care nurses. Home-care nurses are often the first health-care professionals to enter the home and therefore the first professionals to respond to the situation. Our results are valuable to nurses in many other countries with similar contexts and experiencing similar challenges. Another strength is that our study underlines the importance of integrated person-centred care, irrespective of the diseases, to help very vulnerable patients who are in danger of not getting the care they need.

A possible limitation is the convenience sample of people with practical experience of patients with dirty homes. The researchers found no new information in the final interviews and concluded that data saturation was reached. However, there is no way to know whether the full range of problems and solutions was covered, due to the many influencing factors. The problems the individual interviews revealed were considered very recognizable when the possible solutions were discussed in the second round of interviews and four focus groups, indicating that the findings are valid and generalizable. Also the four focus groups suggested similar solutions, indicating that the solutions are valid and generalizable.

## Conclusion

This study showed that home care for patients with a dirty home is complex and home-care nurses experience a variety of problems. An integrated person-centred care approach is often necessary and home-care nurses need extra resources to provide such care. These resources relate not only to the home-care nurses themselves; the management of the home-care organization needs to provide support for their personnel and to organize collaboration between organizations in the community.

## Data Availability

The datasets generated and/or analysed during the current study are not publicly available due to confidentially concerns and/or because full de-identification will possibly lead to misinterpretation. Data are available from the corresponding author on reasonable request.

## Abbreviation

DGPR: General Data Protection Regulation
